# A Sero-epidemiological Study of Arboviral Fevers in Djibouti, Horn of Africa

**DOI:** 10.1371/journal.pntd.0003299

**Published:** 2014-12-11

**Authors:** Fred Andayi, Remi N. Charrel, Alexia Kieffer, Herve Richet, Boris Pastorino, Isabelle Leparc-Goffart, Ammar Abdo Ahmed, Fabrice Carrat, Antoine Flahault, Xavier de Lamballerie

**Affiliations:** 1 Aix Marseille Université, IRD French Institute of Research for Development, EHESP French School of Public Health, EPV UMR_D 190 “Emergence des Pathologies Virales”, Marseille, France; 2 IHU Méditerranée Infection, APHM Public Hospitals of Marseille, Marseille, France; 3 Ecole des Hautes Etudes en Santé Publique de Rennes (EHESP), Sorbonne Paris Cité, Rennes, France; 4 French National Reference Centre for Arboviruses, IRBA, Marseille, France; 5 Ministère de la Santé, Institut National de Santé Publique, Djibouti, Republic of Djibouti; 6 INSERM UMR-S 707, Université Pierre et Marie Curie, Paris 6; Department of Public Health, Hôpital Saint-Antoine, AP-HP, Paris, France; Centers for Disease Control and Prevention, United States of America

## Abstract

Arboviral infections have repeatedly been reported in the republic of Djibouti, consistent with the fact that essential vectors for arboviral diseases are endemic in the region. However, there is a limited recent information regarding arbovirus circulation, and the associated risk predictors to human exposure are largely unknown. We performed, from November 2010 to February 2011 in the Djibouti city general population, a cross-sectional ELISA and sero-neutralisation-based sero-epidemiological analysis nested in a household cohort, which investigated the arboviral infection prevalence and risk factors, stratified by their vectors of transmission. Antibodies to dengue virus (21.8%) were the most frequent. Determinants of infection identified by multivariate analysis pointed to sociological and environmental exposure to the bite of *Aedes* mosquitoes. The population was broadly naïve against Chikungunya (2.6%) with risk factors mostly shared with dengue. The detection of limited virus circulation was followed by a significant Chikungunya outbreak a few months after our study. Antibodies to West Nile virus were infrequent (0.6%), but the distribution of cases faithfully followed previous mapping of infected *Culex* mosquitoes. The seroprevalence of Rift valley fever virus was 2.2%, and non-arboviral transmission was suggested. Finally, the study indicated the circulation of Toscana-related viruses (3.7%), and a limited number of cases suggested infection by tick-borne encephalitis or Alkhumra related viruses, which deserve further investigations to identify the viruses and vectors implicated. Overall, most of the arboviral cases' predictors were statistically best described by the individuals' housing space and neighborhood environmental characteristics, which correlated with the ecological actors of their respective transmission vectors' survival in the local niche. This study has demonstrated autochthonous arboviral circulations in the republic of Djibouti, and provides an epidemiological inventory, with useful findings for risk mapping and future prevention and control programs.

## Author Summary

The arboviruses are a group of viruses transmitted by arthropods such as mosquitoes, ticks, or sandflies. These pathogens have complex life cycles and depend on both arthropods and vertebrate hosts for survival and transmission. Recent global increase in cases confirms that they are of great public health concern. In this study, conducted in the winter of 2010, the seroprevalence and determinants of infections were investigated in the republic of Djibouti, Horn of Africa. The highest seroprevalence values were observed for mosquito-borne diseases, in particular dengue (transmitted by *Aedes* mosquitoes); antibodies to dengue virus were found in a fifth of the sampled population. Most Djiboutians were initially unexposed to Chikungunya virus (also transmitted by *Aedes* mosquitoes), but a few months later, many got infected, resulting in an outbreak. Of the few West Nile virus seropositive cases detected, the majority were in places where WNV had been previously identified in *Culex* mosquitoes. In addition, seropositive cases of Toscana-related viruses (transmitted by sandflies), and tick-borne encephalitis virus or Alkhumra-related viruses (transmitted by ticks) were also observed. In this study, the risk of arboviral infections was mostly associated with environmental and behavioural risk factors, with highest risk prevailing in the city centre (District 1). Overall, the results suggest a likely exposure to the local circulation of arboviruses, rather than infections acquired outside the study area. This knowledge, therefore, confirms the impact of arbovirus infections in Djibouti, and is essential for prevention and control programs.

## Introduction

Arboviral fevers are a threat to the global population and warrant a continuous surveillance and monitoring, especially in tropical and subtropical regions, where most of the low income countries are located [1]. Viruses from families of *Togaviridae* and *Bunyaviridae*, and from genus *Flavivirus* are responsible for the majority of human arboviral infection cases. The observed geographical dispersion of arboviral diseases is strongly correlated with the ecological factors and human activities [2]. For example, dengue virus (DENV), Yellow fever (YFV), and Chikungunya (CHIKV) infections tend to spread to all regions where their *Aedes* transmission vectors are present (potentially affecting two thirds of the global human population) [3]. The tick-borne encephalitis virus (TBEV) is endemic in Europe, Russia and Asia in forest, moorland and steppe ecosystems hosting abundant transmission rodent hosts and *Ixodid* vectors. The warm African eco-climates support abundant mammalian hosts, reservoir birds and vectors, which are favourable factors for arboviral transmission [1]. To some extent, the same characteristics apply to the WHO Eastern Mediterranean region (WHO-EMR) [2], [3], to which our study area, Djibouti, belongs. A combination of limited surveillance capabilities for early detection and a lack of routine preventive medicine programs, in part explains why limited information regarding arboviral fevers is available in Djibouti. Nevertheless, the scientific literature provides evidence that essential vectors for arboviral diseases are endemic in the republic of Djibouti. These include some mosquito vectors (*e.g.*, *Aedes*, *Culex* and *Anopheles* species) [4]–[6], ticks (*Ixodes*, *Rhipicephalus*, *Amblyomma*, *Hyalomma species*) [7] and sandflies [8], [9]. In addition, potential animal reservoirs such as nomadic pastoralists' livestock [10], migratory birds [11], and rodents [12], are present. This evidence corroborates the existing risk of outbreaks, since a number of arboviral pathogens have been detected to be in local circulation [4]–[6], [13], [14]. However, the recent information and the associated risk predictors to human exposure are limited or poorly documented. For example, at the time of submission, there were only two reports on Djibouti local causal association of vector transmission to arbovirus: that of mosquitoes vectors to the WNV [15] and DENV [5]. Other reports have either separately documented the vectors of transmission (courtesy of entomological studies) [8], [9] or indirectly documented the detection of arbovirus exposure via biomarkers (courtesy of serological studies) [5], [16]. This study therefore, is an attempt to bridge the existing knowledge gap, based on the Djibouti city general population. It is a cross-sectional analysis nested in a household cohort, which investigates the arboviral infection prevalence and risk factors, stratified by their vectors of transmission. Attention was given to *Culex*- (WNV), *Aedes*- (DENV, YFV and CHIKV), RVF (diverse transmission mechanisms), sandfly- (Toscana (TOSV) and related phleboviruses) and tick- (TBEV and related flaviviruses) borne viruses. The essential purpose was to provide an epidemiological inventory, with useful findings for risk mapping and future prevention and control programs.

## Methods

### Ethics Statement

Households were enrolled into the study after the ethical approval was granted by both this Consortium, which was based at the EHESP French School of Public Health, Rennes France, and the Ethical Review Committee at the *National Institute of Public Health* (INSP) Ministry of Health, Republic of Djibouti. A household was defined as two or more persons staying in the same house, sharing meals and living room space, with or without familial relationship [17]. For a household to be enrolled, all subjects belonging to it were required to give a written consent before participation. Minors below 18 years were to give their consent through their parents or guardians. This consent also provided for specimen usage in other studies, apart from the CoPanFlu program. This was a Djibouti cohort of pandemic influenza (CoPanFlu) study that investigated the sero-epidemiology and vaccination intention of 2009 pandemic influenza (H1N1pdm09) in the republic of Djibouti [18]. The study was based on the WHO-EHESP CoPanFlu International Consortium core protocol [19].

### Study Area, Djibouti City

**Figure 1:**
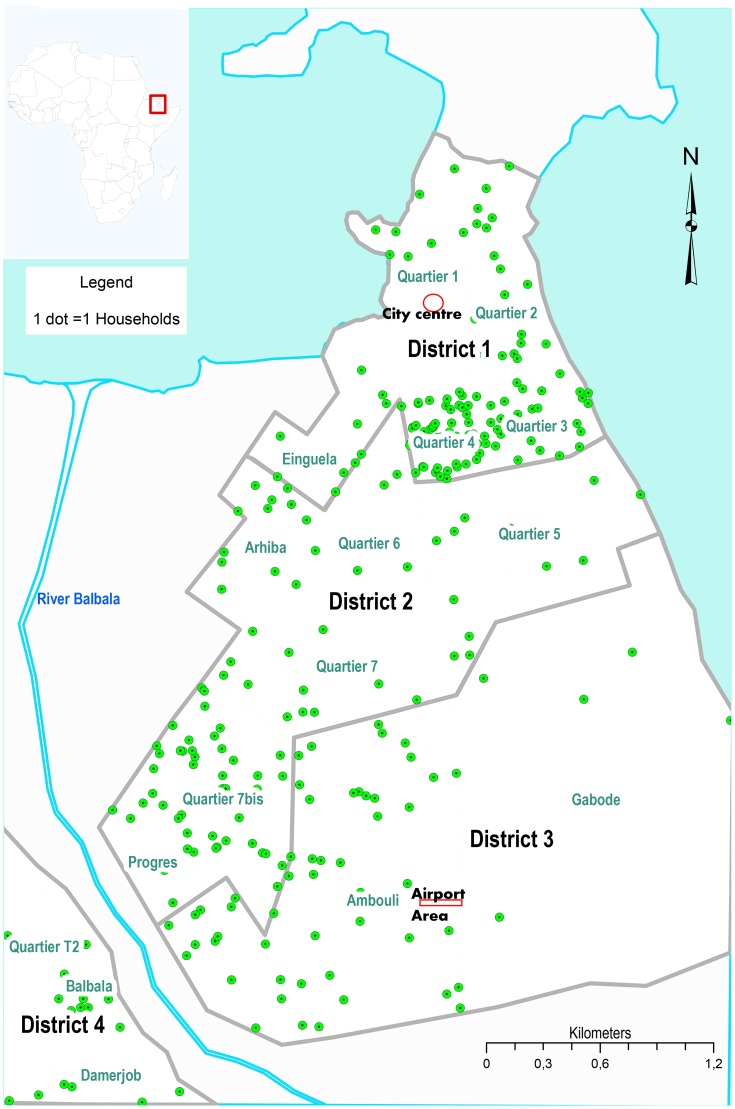
A map of the study area, Djibouti city, horn of Africa, showing the spatial distribution of households by Quartier(location) in the four administrative districts.

The study was conducted in four administrative districts of Djibouti city, Republic of Djibouti, which is one of the 22 member states of the WHO Eastern Mediterranean region [20]. It is situated in the horn of Africa, at the Gulf of Eden of the Red Sea, bordering Somalia, Ethiopia, and Eritrea. It covers 23,200 km^2^ with 818,159 inhabitants, with majority of them, 70.6% (577,933) residing in urban areas [21]. Of those who live in urban, the largest proportion, 58.1% (475,322) are inhabitants of the capital, Djibouti city. Eco-geographically, the country is largely arid and semi arid, with perennial flooding during winter (November to April) and prolonged summers for the rest of the year. Fig. 1 shows an illustrative map of the study area, Djibouti city, together with the spatial distribution of participating households by Quartier (location) in the four administrative districts. The District 1 hosts the city center and there is a progressive decline in the urbanization, from District 2 towards District 4.

### Study Design (Based on CoPanFlu Djibouti Program)

The protocol and samples used in this study were derived from the Djibouti Cohort of Pandemic Influenza (CoPanFlu) program [18], [19], as mentioned above. After receiving authorization from relevant government departments, 1,045 individuals from 324 households were enrolled randomly, between 11^th^ November 2010 and 15^th^ February 2011, from a pool of 1,835 households, which were derived from two sources: 1,335 households were from the 2009 Hajj Pilgrim database and 500 households were from the community of health workers (CHW) cognisance list of vulnerable households. The initial project was designed as a seroprevalence study and therefore no specific clinical information was used for recruitment. Complete households were included, whatever the medical history of each family member. Information was given to household members and enrollment was conducted only when all members could be included. Participants or their legal representatives were “*a priori*” required to give informed consent. Only households meeting the following criteria were enrolled in our cohort: all members of the household shared one roof, they shared meals and living area, and consented to participate (including blood sampling and responding to questionnaires). On an appointed date, the capillary blood samples (∼100–500 µL) were collected and the assisted response to standardised French questionnaires was completed, using the local dialect to translate questionnaires whenever necessary. This questionnaire collected information on subjects' and households' profiles, occupation and academic background, and residential environment characteristics (see **Table 1** for details). Because of the initial purpose of the cohort, information relating to yellow fever vaccination was not collected. After completion of the CoPanFlu program, the current study was performed using the biological samples that remained available, *i.e.*, those of 1,045 subjects recruited in 324 households. The spatial distribution of the enrolled households by Quartier (location) is illustrated in **Fig. 1** [18].

**Table 1:** The Djiboutian cohort demographic characteristics and study variables (N = 1,045).

(a). Subject and household profile	n	%	(b). Residential environment	n	%	(c). Occupation and literacy	n	%
Age group: ≤19 years	409	39.1%	Residential District: 1	433	41.4%	Occupation: ≤13years	109	10.4%
20–39 years	435	41.6%	2	340	32.5%	Employed	162	15.5%
40–59 years	155	14.8%	3	200	19.1%	Jobless	473	45.3%
≥60 years	36	3.4%	4	72	6.9%	Student	301	28.8%
Unknown	10	1.0%	Living nearby riverbank:	45	4.3%	Education: ≤5years	88	8.4%
Gender: Women	571	54.6%	Living nearby dumpsite:	140	13.4%	Illiterate	265	25.4%
Men	474	45.4%	Living nearby food market:	410	39.2%	Basic education	201	19.2%
Family size: Small (≤3 persons)	563	53.9%	Living nearby vegetable market:	308	29.5%	Bac+	123	11.8%
Medium (4 or 5 persons)	297	28.4%	Living nearby abattoir:	281	26.9%	Unknown	368	35.2%
Large (≥6 persons)	185	17.7%	Living nearby open sewage:	24	2.3%	Working from outdoors:	77	7.4%
Children in household: none	290	27.8%	Sleeping out in open at night:	75	7.2%	Working from indoors:	96	9.2%
Few (≤3)	650	62.2%	Keeping animal(s):	149	14.3%			
More (4≥)	105	10.0%	Exposure to birds:	11	1.1%			
Ethnicity: Afar	101	9.7%						
Arab	174	16.7%						
Ethiopian	58	5.6%						
Migrants	15	1.4%						
Somalis	645	61.7%						
Unknown	52	5.0%						
SES Level: Low	285	27.3%						
Middle	127	12.2%						
Upper	392	37.5%						
Unknown	241	23.1%						

### Laboratory Analyses

The screening of antibodies (IgG) against various pathogens was performed using two different Enzyme Linked Immuno-Sorbent Assay (ELISA) protocols. In the first protocol, in-house kits (in which antigen derived from whole-virion particles in non-inactivated cell culture supernatants) were used to test for YFV, TOSV, RVFV and CHIKV antibodies. In the second protocol, commercial kits were used for detection of DENV (PanBio, Brisbane, Australia), WNV and TBEV (EuroImmun, Lübeck, Germany) antibodies. Positive and negative control sera were provided by the *French National Reference Centre for Arbovirus* or by the kits' manufacturers. For each serologic assay, a minimum of three positive controls was included, alongside three negative controls and three blank controls (normal saline), in accordance with the established standard protocols [22]. Additional sero-neutralisation experiments were conducted in which wild-type laboratory-adapted viral strains were used, with exception of the YFV, in which the D17 vaccine strain was used. Appropriate cell culture lines and reagents were used in accordance to the established Standard Operating procedures and Good Laboratory Practice protocols of the laboratory. All experiments were conducted in *Biosafety level 3* laboratory containment facilities, at the EPV UMR_D 190 research laboratory, or at the French National Reference Centre for Arboviruses, Marseille France.

### ELISA Protocol

#### In-house kits

Onto Maxisorp 96 well plates (Nunc), a 100 µL per well of 1∶200 of virus supernatant at 10^5^–10^7^ pfu per ml, in PBS buffer, at pH 9.6, was added and incubated overnight at 4°C. The supernatant was discarded, and the plates blocked with 300 µL of PBST-10% milk (containing 0.05% tween-20 (v/v), 10% non-fat dried milk (w/v) and PBS) and incubated at 4°C for 90–120 minutes. The plates were then washed thrice with phosphate-buffered saline (PBS; pH 7.4 and 0.05% tween-20 (v/v)). Afterward, a 1∶200 of test sera in PBST-5% milk was added in duplicate wells and incubated for 60 minutes at 37°C. The plates were washed as before, followed by the addition of 100 µL of a 1∶8000 dilution of goat F(ab′)2 fragment anti-human IgG(H+L) peroxidase (Beckman Coulter) in PBST-5% milk, and incubated for 90 minutes at 37°C. Plates were washed six times and a 100 µL TMB substrate (SureBlue) added to develop the reaction. This reaction was terminated by addition of 100 µL Stop solution (1M Hydrochloric acid) after 30 minutes. The absorbance was read in a microplate ELISA reader (Bio-Rad Benchmark) at 450 nm.

#### Commercial kits

ELISA was performed according to the manufacturer's instructions and the optical density (absorbance) was read in a microplate ELISA reader machine (Bio-Rad Benchmark) at 450 nm.

### ELISA Screening Cutpoint Determination

For consistence, all samples were tested in duplicates using common serum controls (negative and positive) for all plates in a specific pathogen assay. The values of all plates for a given test were subsequently normalised according to values of negative and positive controls. In addition, a panel of 176 true negative samples was tested using the in-house and commercial kits protocols. This panel included sera from a previous study of French blood donors that tested negative for antibodies to all pathogens studied here using sero-neutralisation techniques. For both in-house and commercial assays, sera with normalised absorbance values above the cut-off value (defined as [mean of normalised true negatives+two standard deviations]) were considered to be positive. The positivity ratio (normalised absorbance value of the sample/cut-off) was used for ELISA interpretation with ratios ≤0.9 associated with negative results; ratios between 0.9 and 1.1 with equivocal results; and ratios ≥1.1 with positive results.

### Micro-neutralisation Assay Protocol

A virus neutralisation assay (VNT) was performed for all viruses, but dengue to check the performance of the ELISA assays. In brief, 50 µL of heat-inactivated (56°C, 30 minutes) serum dilution (5 to 1280 in PBS) was added to 50 µL of viral suspension (representing 100 TCID_50_) in flat bottomed 96-well cell culture Microplates (Nunc) followed by 100 µL of Vero cell suspension (2×10^5^ cells/ml) in MEM culture medium supplemented with 8% fetal bovine serum and antibiotics. The plates were then incubated at 37°C in CO_2_ incubator and virus multiplication was measured after 3–5 days by observing a cytopathic effect (CPE) or by quantifying the amount of viral genome in the culture supernatant by using real-time RT-PCR techniques in the case of TBEV and Alkhumra virus (AHFV) (below is the protocol). Absence of CPE or real-time RT-PCR cycle threshold above 37 was considered a positive reaction. The final arbovirus infection status (seropositivity) of the subjects was determined by the VNT positive status, which was set at a cutpoint titer of ≥10 [23].

### Quantitative Real-Time Reverse-Transcription PCR (QRT-PCR)

The TBEV ELISA seropositives samples were tested for neutralising antibodies against TBEV and AHFV in the VNT assay. For each, the VNT culture was used for RNA extraction for TBEV and AHFV qRT-PCR assay. The RNA extraction was performed using the NucleoSpin 96 RNA virus kit (Macherey-Nagel) in accordance to the manufacturer's instructions. The TaqMan NS3 primers and probes sequences used for amplification were as follows: ALKV-Forward (CCA GTT GTY TCC ATG GAT GG), ALKV-Reverse (GCC GCC AAC CQA CAQ TGG) and ALKV-Probe (FAM-CAA TGT AGC TAG CCT GAT AAC T-TAMRA); TBEV-Forward (GGA MGR ACM GAT GAA TAC AT), TBEV-Reverse (GYG CYT CYT TCC AYT GCA) and TBEV-Probe (CTC TGG ACA GTG TGA TGA TGA TGA). The probes were labeled with fluorescent 6-carboxyfluorescein (FAM) as the reporter dye at the 5′ end and with a quencher of the minor groove binder (MGB) at the 3′ end. The PCR reaction kit constituted of the one-step SuperScript III Platinum QRT-PCR System with Rox (Invitrogen) and the reaction was performed in the Applied Biosystems 7900 Real-Time PCR System. A total of 20 µL reaction volume was used, consisting of 0.5 µL of Superscript III RT/Platinum Taq Mix, 10 µL of Reaction mix with ROX, 0.5 µL of Reverse primer, 0.5 µl of Forward primer, 3 µL RNA template, 0.3 µL of the probe and 5.2 µL of water. The reaction condition entailed 60°C for 15 minutes for RT, 95°C for 2 minutes and 40 cycles of amplification (95°C for 30 sec; 60°C for 30 sec). Wells with cycle threshold lower than 37 were considered to have a negative result for neutralising antibodies, otherwise were considered positive.

### Statistical Analysis

Data entry and management was performed in the *FileMaker Pro Advanced 11* (FileMaker) environment. From the 19 household ownership properties, the *principle component analysis* was used to create three socioeconomic status (SES), the Upper SES, the Middle SES and the Lower SES [24]. The criterion used to differentiate the three SES levels was based on rank score of household property ownership, and as described in details elsewhere by Vyas *et al.* [24] and Nauta [25]. The 19 household properties used for SES determination, included the ownership of *Vehicle*, *Music System*, *Washing Machine*, *Sealing Air Fan*, *Bicycle*, *Toilet*, *Telephone*, *Television*, *Separate Seating/Lounge Room*, *Radio*, *Motor Cycle*, *House Owner*, *Fridge*, *Electricity*, *DVD Video Player*, *Gas Cooker*, *Electric Cooker*, *Air Conditioner System*, *and running tap water*. In some cases, this information was missing and therefore the SES level was documented as ‘unknown’ (**Table 1**). A descriptive analysis was performed on variables in preliminary evaluation. For public health importance, the infection status (seropositivity) was stratified for analyses as (a) *individual pathogens* or according to their (b) *transmission vectors*, namely: *Culex*-borne viruses (WNV), RVF (diverse transmission mechanisms), *Aedes*-borne viruses (YFV, DENV and CHIKV), sandfly-borne viruses (TOSV) and tick-borne viruses (TBEV and AHFV); or (c) *virus taxonomy*, namely: *Flaviviruses* (DENV, WNV, TBEV, AHFV, YFV), *Phleboviruses* (RVF and TOSV) and *Togaviruses* (CHIKV). Evaluation of heterogeneity of the seropositivity proportions in different independent variables such as districts, age groups, occupation and SES, was done by Chi square (χ^2^) test or Fisher's exact test. Analysis of trend to establish the potential systematic increase or decrease of infection status across the variable was also performed. At the time of study, no specific information regarding specific exposure risk to the different vectors was available to the authors. Therefore the determination of socio-demographic and environmental predictors to infection status were performed in the generalised estimating equation (GEE) models, which accounted for the household clustering effect among the enrolled subjects. Variables with p-value ≤0.25 in bivariate model were included in multivariate analysis in a backward stepwise reduction protocol, those with p-value ≤0.15 were retained in the final model and those with p-value ≤0.05 being considered statistical significance [26]. Effect modification and interaction between variables on subjects' seropositivity were assessed. The use of GEE model in measurement of association, did not allow for the institution of post estimation validity evaluation [27]. All analyses were conducted in *Stata Statistical Software Release 13* (StataCorp College Station, TX: StataCorp LP).

## Results

Demographic information for the 1,045 subjects belonging to 324 families involved in this study is shown in **Table 1**, a detailed profile has been provided elsewhere [18]. Briefly, the participants were drawn from different age groups, gender, residential districts, ethnicity, occupation and socio-economic background in Djibouti city. Their diversity was manifested also in living conditions (housing space) and neighborhood environment, which included: housing materials, domestication of animals, exposure to birds, sleeping habits (out in the open at night), and the proximity to the following: market, abattoir, open sewage, dumpsite and river bank, respectively. The performance of the different ELISA tests was examined with reference to sero-neutralisation results for all viruses studied except DENV. Due to capillary sampling, there was limited amounts of serum and therefore we could not perform more biological tests on DENV (and some ELISA positive subjects) than those described in the article. However, from past studies [5], DENV circulation had been broadly recognised in Djibouti, and there was little doubts that DENV represented the most frequent arbovirus transmitted. But for the rest, for each test, a selection of ELISA negative samples and all available samples with a positive ELISA result were tested.

For those viruses which had been previously identified in East Africa (YFV, WNV, CHIKV and RVFV) results are summarised hereafter and available in **Table 2** and **Table 3**: *(i)* for YFV, 11 out of the 14 ELISA-positive samples were available for VNT. The Positive Predictive Value (PPV) of the ELISA test (ratio ≥1.1) was 0.64 and the Negative Predictive Value (NPV, calculating after gathering negative and equivocal results) was 1. We therefore tested other ratios for the definition of positives and identified an optimised ELISA ratio at 1.5, associated with a PPV at 0.91 and a NPV at 0.88. *(ii)* For WNV, 4 out of the 5 ELISA-positive samples were available for VNT. The ELISA PPV (ratio ≥1.1) was 0.56 and the NPV was 1; an optimised ELISA ratio at 1.3 was associated with a PPV at 0.75 and a VPN at 0.80; *(iii)* for CHIKV, 23 out of the 24 ELISA-positive samples were available for VNT. The ELISA PPV (ratio ≥1.1) was 1 and the NPV was 0.86; *(iv)* for RVFV, 18 out of the 20 ELISA-positive samples were available for VNT. The ELISA PPV (ratio ≥1.1) was 0.83 and the NPV was 1.

**Table 2:** A matrix of various arboviruses sero-positivity profile for residents of Djibouti city in the winter of 2010.

Arboviral pathogens (tested samples)	ELISA positive cutpoint	VNT/ELISA Positives	ELISA seropositivity profile: Number of arboviruses tested positive		Specific virus ELISA seropositivity profile						
			1	≥2	CHIKV	TOSV	RVF	YFV	WNV	TBE	DENV
**CHIKV (914)**	1.1	23/24	7	15	7	2	1	0	0	0	17
**TOSV (915)**	1.1	30/34	15	9	2	15	9	6	0	0	11
**RVF (914)**	1.1	15/20	8	5	1	9	8	7	1	0	3
**YFV (903)**	1.5	10/14	4	4	0	6	7	4	1	0	4
**WNV (893)**	1.3	3/5	2	3	0	0	1	1	2	0	1
**TBE (893)**	1.1	1/5	5	0	0	0	0	0	0	5	0
**DENV (911)**	1.1	n/a	160	23	17	11	3	4	1	0	160

**Table 3:** Prevalence of neutralizing anti-arbovirus antibodies among Djibouti population in the 2010 winter period.

Arboviral Pathogen	ELISA Positives		VNT tested samples*	Micro-neutralisation assay titers					VNT Positives (titre ≥10)	Total Subjects (N = 1,045)
	Cut point (≥)	Count		5	10	20	40	>80		
DENV	1.1	199	-**	-	-	-	-	-	-	911
TBEV	1.1	5	2	1	0	1	0	0	1	893
AHFV	n/a***	-	3	1	1	0	1	0	2	893
RVFV	1.1	20	18	3	7	4	4	0	15	914
CHIKV	1.1	24	23	0	5	6	7	5	23	914
TOSV	1.1	34	32	2	5	16	6	3	30	915
YFV	1.5	14	11	1	4	4	2	0	10	903
WNV	1.3	5	4	1	1	2	0	0	3	893

*Elisa positive samples,

**Insufficient samples for VNT assay,

***TBEV Elisa positivesamples.

For viruses never isolated in the region, the results were as follows: *(i)* for TOSV, 33 out of the 34 ELISA-positive samples were available for VNT. The ELISA PPV and NPV (ratio ≥1.1) were 0.94 and 0.90, respectively, suggesting previous contact with genuine TOSV or a closely related phlebovirus (see below). *(ii)* for TBEV, all of the 5 ELISA-positive samples tested negative for other flaviviruses tested (DENV, YFV, WNV), which rules out the hypothesis of cross-reactivity with one of these pathogens. Since only two samples were available for additional seroneutralisation tests, reliable PPVs could not be calculated. The first sample was negative in seroneutralisation for TBEV and also for AHFV, a tick-borne flavivirus that has been previously shown to circulate in Saudi Arabia and in the south of Egypt [28], [29]. The second sample was positive for TBEV (titre 20) and AHFV (titre 40), suggesting possible contact with the latter virus. The sample was from a 13 yo girl belonging to a family with a low socioeconomic status, and living nearby an abattoir. Her age and socioeconomic status make unlikely a previous travel to known AHFV endemic areas such as Saudi Arabia (*e.g.* for the Hajj).

Using the aforementioned optimised ELISA positivity criteria, mosquito-borne virus infections were predominant, with 23.6% of the population testing positive for at least one *Aedes*-borne virus (DENV, YFV and/or CHIKV) and 0.6% to WNV; 3.7% tested positive for sandfly-borne viruses (TOSV); 0.6% tested positive for tick-borne viruses (TBEV); 2.2% tested positive for RVFV. Detailed results are provided in **Tables 2** and **Table 3**.

All subsequent statistical analyses were performed using ELISA results (with reference to optimised positivity ratios).

First, we analysed double-positives (*i.e.*, samples with positive results for two different tests) and the issue of possible ELISA antigenic cross-reactivity between the different flaviviruses tested (DENV, YFV, WNV and TBEV) was addressed. No significant statistical association between serological results for flaviviral species was observed, confirming the good PPV of the ELISA tests for most of flaviviruses tested. The same analysis was performed for phleboviruses and a strong association was observed (p<0.00001) between TOSV and RVFV results. This is intriguing since TOSV and RVFV are antigenically very distant. A refined analysis of ELISA results for double-positives identified no relationship between the positivity ratios of TOSV and RVFV ELISA positives. Moreover, in double-positives, VNT geometric mean titers (GMTs) were high for both viruses (>20 for RVFV, >30 for TOSV). Altogether, this suggests that an epidemiological relationship rather than an antigenic cross-reactivity should be invoked.

Second, we examined possible associations that might be explained by exposure to a common vector. An obvious association (p<0.00001) was identified between DENV and CHIKV (70.8% of CHIKV positive samples are also DENV positive). This is evocative of a shared exposure to the bite of *Ae. aegypti*, which represents the most probable vector of both CHIKV and DENV. The same link was not identified between YFV and CHIKV or DENV, despite their common vector. However, since YFV is not endemic in Djibouti, the most probable explanation to YFV positive results is either immigration from an endemic country or vaccination (as recommended for Hajji Pilgrims and for travels in Ethiopia, see below).

Third, a triangular significant association (p<0.00001) was observed between YFV, TOSV and RVFV. Since there is no antigenic relationship between YFV and phleboviruses, and different vectors transmit all three pathogens, the existence of a subpopulation gathering a variety of risk factors represents the most plausible explanation (see supplemental data in **S1 Table**).

Univariate and –when authorised by numbers– multivariate analyses (UVA and MVA, respectively) were performed to assess the relationship between serological results to subject and household profiles', occupation and academic background, and the residential environment characteristics (see **S2 Table** and **Table 4**). Statistical analysis indicated that DENV and CHIKV positives share a number of risk factors: *(i)* living in District 1 (*i.e.*, city centre; MVA, p<0.001) and sleeping outside at night (MVA, p<0.001). Sleeping out is a common practice among those families with no air conditioner machine. This is because during the summer period in Djibouti, the ambient temperature and humidity are in the extreme. This often compels them at night, to literally sleep outside their houses, in the open air, many of whom, without bednets, so as to catch some sleep. Both (CHIKV and DENV) are most probably linked with exposure to the common vector (*Ae. aegypti*), which is likely, in the warm and dry Djiboutian setting, to find a favourable environment in the urban areas of Djibouti, as previously reported in other locations [30] and can bite in the evening and the beginning of the night. *(ii)* living in large families (four or more persons; MVA, p<0.001). This may reflect favourable conditions for maintaining a population of *Aedes* mosquitoes in/around the household. In accordance with this observation, MVA indicates that keeping a domestic animal at home (which indeed may be part of the feeding resources available for female *Aedes* mosquitoes) is also associated with an increased risk for dengue (MVA, p<0.001). Amongst residential environment parameters, living nearby a river was associated with an increased risk for dengue (MVA, p<0.001). Running water is seasonal in Djibouti and, in the extreme context of the local climate, which is associated with limited vegetation, the presence of plant cover, puddles and water holes on the river banks is likely to offer an alternative to urban households as a source of larval sites for *Aedes* mosquitoes. Living nearby meat-markets (mostly located in peripheral poorly urbanised areas; MVA, p<0.001) and having a high socioeconomic status (MVA, p<0.001) were found to be protective for DENV and CHIKV, respectively. It is important to note that these findings do not account for the population dynamism within and without the Djibouti city administrative districts.

**Table 4:** Determinants of arbovirus sero-positivity among Djibouti city residents in the winter of 2010.

Risk factors for Dengue virus	aOR*	95% LCL	95% UCL	pvalue	Risk factors for Chikungunya virus	aOR	95% LCL	95% UCL	pvalue
*(a) Subject and household profile*					*(a) Subject and household profile*				
Age group: ≤19 yo	0,7	0,5	1,0	**0,0290** **	Gender: woman	0,6	0,3	1,3	0,1920
20–39 yo	Ref	Ref	Ref	Ref	Age group: 20 to 39 yo	1,9	0,9	4,3	0,1020
40–59 yo	0,7	0,5	1,1	0,1340	Large family: 6 or more persons	5,7	2,6	12,5	**<0,0001**
≥60 yo	1,2	0,7	2,0	0,4940	Ethnic: Afar	2,0	0,8	5,0	0,1180
Gender: woman	0,8	0,6	1,1	0,1230	SES status: Upper	0,3	0,1	0,7	**0,0050**
Large family: 6 or more persons	1,7	1,3	2,2	**<0001**	Lower	0,4	0,1	1,2	0,1100
*(b) Occupation and literacy*					*(b) Occupation and literacy*				
Employed:	0,7	0,4	1,0	**0,0330**	Employed:	0,2	0,0	1,5	0,1220
Students:	0,6	0,4	0,8	**0,0020**	Illiterate:	0,4	0,1	1,3	0,1200
Illiterate:	0,7	0,5	0,9	**0,0170**	*(c) Residential environment*				
*(c) Residential environment*					Residential District: 1	8,2	2,8	23,9	**<0,0001**
Residential District: 1	1,7	1,3	2,2	**<0001**	Sleeping out in open at night:	3,4	1,3	8,4	**0,0100**
Living nearby river bank:	2,3	1,5	3,4	**<0001**					
Living nearby dumpsite:	0,5	0,3	0,8	**0,0100**	**Risk factors for Toscana virus**	aOR	95% LCL	95% UCL	pvalue
Living nearby abattoir:	0,4	0,3	0,6	**<0001**	*(a) Subject and household profile*				
Sleeping out in open at night:	2,8	1,8	4,3	**<0001**	Age group: ≤19 yo	1,3	0,6	2,8	0,4470
Keeping animal(s):	1,9	1,4	2,5	**<0001**	Gender: woman	1,4	0,7	2,9	0,3050
					Ho usehold with children:	1,6	0,6	4,2	0,3200
**Risk factors for Yellow fever virus**	aOR	95% LCL	95% UCL	pvalue	*(b) Occupation and literacy* ** = None = **				
*(a) Subject and household profile*									
Age group: ≤19 yo	2,3	0,7	7,3	0,1540	*(c) Residential environment*				
Gender: woman	1,5	0,5	4,7	0,4800	Living nearby dumpsite:	2,0	0,9	4,6	0,1000
Large family: 6 or more persons	2,5	0,8	8,0	0,1200	Residential District: 1	3,0	1,4	6,3	**0,0040**
SES status: Upper	10,1	1,3	80,6	0,0290					
Middle	9,5	0,9	104,4	0,0650	**Risk factors for Rift Valley Fever virus**	aOR	95% LCL	95% UCL	pvalue
(*b) Occupation and literacy*					Gender: woman	1,1	0,5	2,7	0,8040
Employed:	1,3	0,1	20,5	0,8350	Ethnic: Arab	3,4	1,3	9,2	**0,0160**
Working from indoors:	5,3	0,7	43,3	0,1190	Ethnic Arab & of Upper SES:	0,2	0	1,5	0,1190
Student:	2,7	0,2	29,4	0,4260	Age group: ≤19 yo	3,4	1,3	8,9	**0,0150**
*(c) Residential environment*					Large family: 6 or more persons	2,1	0,8	5,5	0,1220
Living nearby food market:	1,1	0,2	5,2	0,8720					
Living nearby Vegetable market:	0,2	0,0	2,0	0,1660					

*, adjusted odd ratio from multivariate analyses,

**, statistically significant association at pvalue ≤0.05 are bolded.

Finally, the distribution of sero-prevalence in age groups showed that the highest incidences were in young adults (20–39 yo) for both DENV (22.2%) and CHIKV (3.0%), see **Table 5**. This profile suggests that DENV virus circulation in Djibouti is not under a regimen of hyper-endemicity as observed in Southeast Asia; here, in contrast, it is suggestive of probable episodic spillovers into a population that was broadly naïve in all age groups. An important consequence is that dengue is not a specifically paediatric disease in Djibouti.

**Table 5:** Prevalence of anti-arboviral IgG antibodies by administrative district of Djibouti city, age group and gender in the winter of 2010 (N = 1,045).

Arboviral Pathogens	Sample tested (N = 1045)	Positive cases (x = 251)	Overall prevalence (%)	Gender		Age group (years)				Residential District			
				Male	Female	≤19	20–39	40–59	≥60	1	2	3	4
				n = 466	n = 571	n = 409	n = 435	n = 155	n = 36	n = 433	n = 340	n = 200	n = 72
				(%)	(%)	(%)	(%)	(%)	(%)	(%)	(%)	(%)	(%)
**DENV**	911	199	21.8	20.0	18.4	15.9	22.1	17.4	30.6	25.9	13.8	13.5	18.1
**WNV**	893	5	0.6	0.4	0.5	0.5	0.5	0.6	0.0	0.7	0.0	0.5	1.4
**TBE**	893	5	0.6	0.4	0.5	0.7	0.0	1.3	0.0	0.2	0.9	0.5	0.0
**YFV**	903	14	1.5	1.5	1.2	2.2	0.9	0.6	0.0	1.4	1.8	0.0	2.8
**CHIKV**	914	24	2.6	2.6	2.1	2.0	3.0	0.6	5.6	4.6	0.6	0.5	1.4
**RVF**	914	20	2.2	2.1	1.8	3.4	0.7	1.9	0.0	2.1	2.1	1.5	1.4
**TOSV**	915	34	3.7	3.0	3.5	3.9	3.0	2.6	2.8	4.6	2.6	1.0	4.2
**Any of the arboviruses**	916	251	27.4	25.3	23.1	22.2	26.2	22.6	30.6	31.6	19.1	16.5	22.2
***Flaviviruses***	915	217	23.7	21.7	20.1	17.8	23.4	20.0	30.6	27.3	16.2	14.5	20.8
***Phleboviruses***	915	45	4.9	4.5	4.2	5.6	3.4	3.9	2.8	5.5	3.5	2.5	5.6
***Alphaviruses***	914	24	2.6	2.6	2.1	2.0	3.0	0.6	5.6	4.6	0.6	0.5	1.4
**Tick-borne**	893	5	0.6	0.5	0.6	0.9	0	1.4	0	0.3	1.0	0.6	0
**Aedes-borne**	910	215	23.6	25.0	22.5	21.7	26.1	20.0	31.4	34.7	16.8	14.3	25.0
**Culex-borne**	893	5	0.6	0.4	0.5	0.5	0.5	0.6	0.0	0.7	0.0	0.5	1.4
**Sandfly-borne**	915	34	3.7	3.0	3.5	3.9	3.0	2.6	2.8	4.6	2.6	1.0	4.2

Regarding CHIKV, these numbers indicate that when this study was performed (November 2010 to February 2011), the Djiboutian population was massively naïve towards Chikungunya. The O'nyong-nyong (ONNV) and CHIKV are the two alphaviruses previously observed in the region [31], [32], with potential antigenic cross-reactivity. However, the measure of ONNV specificity to CHIKV in immuno-fluorescence and haemagglutination inhibition techniques gave limited (if any) cross reaction using monoclonal antibodies [31]. This was consistent with Chanas *et al.* [32] observations, that the antibodies to ONNV are quite specific and poorly seroneutralise CHIKV. Therefore, we are confident that we detected mostly antibodies to CHIKV because: *(i)* we used non-inactivated viral antigen that allows to favour a specific selection of antibodies to envelope glycoproteins, which are the most divergent antigens between the two species, and *(i)* our ELISA results were broadly confirmed by seroneutralisation.

Regarding other viruses, the low sero-prevalence rates did not allow to identify major risk factors. *(i)* In the case of TBEV, UVA suggested migrants as a target population (p = 0.01), which may reflect specific exposure to tick bites, presumably through long periods of time spent in a rural environment and/or contact with livestock. In the case of WNV, children under the age of 13, not sent to school nor employed appeared to be at risk (UVA, p = 0. 01), possibly reflecting low socioeconomic status. *(ii)* In the case of YFV, no strong correlate was identified. *(iii)* In the case of TOSV, positives were more frequent in District 1 (MVA, p = 0.004), and this may guide future investigations for identifying the vector and deciphering the transmission cycle in Djibouti. *(iv)* In the case of RVFV, MVA identified an elevated risk of infection amongst the young below 19 yo (p = 0.0150) and individuals of Arab descent (p = 0.0160). Of note was that about a half (48.3%) of those in upper SES class were of Arabian ethnicity, and that the young Arabs were at least risk compared to their contemporaries (although not statistically significant). This may reflect a link with animal sacrifice related activities during Islam feasts or travel in countries of high endemicity, since upper SES Arabian adult populations could afford such foreign trips, compared to majority of other tribes [33]. Regarding triple exposure to TOSV, RVFV and YFV, the best correlates identified by MVA were the age under 19 yo (p = 0.02) and the Afar ethnic origin (p = 0.02), possibly reflecting specific risk factors such as transhuman pastoralism, contact with livestock (this age group is commonly in charge of the animals) and travels in Ethiopia (for which YF vaccination may be required) since the largest Afar populations reside in the Danakil Desert in Ethiopia.

## Discussion

This study reports arboviral sero-prevalence values and risk predictors in the winter of 2010, in Djibouti city. Of the total participants, over a quarter (27.4%) had evidence of infection with at least one of the eight studied arboviruses. Studying simultaneously a variety of pathogens allowed us to weight serological cross-reactions. With reference to sero-neutralisation assays, it was minimal, reflecting our choice of giving priority to the Positive Predictive Value of the tests used (with the possible consequence of slightly under-estimating actual prevalence rates).

Of interest was the conspicuously high burden of mosquito-borne viruses, especially, those transmitted by *Aedes* mosquitoes. DENV was found to have the largest and with widest distribution across the different residential Districts of the city. It was first reported in the outbreak of 1991–1992 [5], then remained steadily in circulation and was subsequently detected in survey studies [14]. So far DENV serotypes 1, 2 and 3 are documented to have circulated in Djibouti [5], [14]. whereas the presence of DENV Serotype 4 has not been reported.

Our results suggest that dengue is present but still circulating at low levels compared with countries of high endemicity, resulting in limited immune protection of the population and infections distributed in age groups (*i.e.*, not predominantly impacting the paediatric population). Determinants of DENV infection identified by multivariate analysis point to sociological and environmental exposure to the bite of *Aedes* mosquitoes.

At the onset of this study, conducted in winter of 2010, contrary to DENV, CHIKV had never been reported in Djibouti. We report here a 2.6% sero-prevalence rate, with epidemiological determinants of infection very similar to those identified for dengue. It is worth noting that a CHIKV outbreak occurred in Djibouti during the year 2011 (personal communication Dr Ammar Ahmed Abdo, Ministry of Health Djibouti) and that, in our study, a majority of individuals with specific antibodies (>80%) were living in District 1. The most probable scenario is therefore that the virus had been circulating at low rate in 2010 in the city centre where exposure to *Aedes* bite appears to be the highest in Djibouti. The epidemic burst occurred in 2011 and this scenario was reminiscent of the Indian Ocean outbreak: CHIKV had been circulating at low level in 2005 in the naïve population of Reunion Island before an impressive burst in 2006 [34], [35]. Therefore, a consideration for the 2011 CHIKV outbreak followup study is desirable, so as to complement and or validate our observations. The predominance of DENV and CHIKV most probably reflects the fact that they are transmitted by the same peri-domestic vector, *Aedes aegypti*, which easily invades, spreads and colonises human habitation [30]. Regarding YFV (also transmitted by *Aedes aegypti*), the low prevalence numbers observed, the absence of epidemiological relationship with other *Aedes*-borne viruses and the lack of reported cases over the last decades in Djibouti suggest the identification of vaccinated individuals rather than the existence of local yellow fever foci.

Unlike *Aedes-*borne viruses, viruses potentially transmitted by *Culex* mosquitoes (WNV) were less represented in this study and no strong risk factors could be identified. The circulation of WNV in Djibouti has been previously documented *(i)* in horses, by the detection of specific antibodies (ELISA followed by PRNT or Western blot) [36] and *(ii)* in mosquitoes, by the molecular detection of WNV genotype 2 RNA in pools of *Culex pipiens* spp. *torridus* and *Culex quinquefasciatus* [15]. Faulde and collaborators identified WNV RNA-positive mosquito pools in site ML4 (airport, positive pools were *Culex quinquefasciatus*) and ML5 (market place, positive pools were *Culex pipiens* spp. *torridus*). Remarkably, of the 5 individuals that tested positive for WNV antibody in the current study, one was living in the ML4 area and three in the ML5 area. Therefore, our results are in accordance with Faulde's findings, but they also confirm the classical discrepancy between the circulation of WNV in mosquitoes and birds and the number of cases of infection in dead-end hosts such as humans and horses (which in addition include a vast majority of asymptomatic or mild cases that do not draw medical attention). The sites ML4 and ML5 are cited from Faulde *et al.* [15] study areas, and correspond to two Quartiers in the current study area (**Fig. 1**), namely, Quartier 1 (City Centre) and Ambouli (Airport area), respectively.

Regarding RVFV, its circulation in livestock has been repeatedly reported in the region [37]–[40]. However, it is noticeable on the one hand, that Djibouti was not in previous studies [38], [39], recognised as a regional hot spot for transmission but a “potential epizootic area”, and on the other hand, that no human case has been reported in Djibouti. In our study, antibody to RVFV was not associated with any epidemiological or environmental parameter that would suggest the implication of mosquitoes. This most probably reflects the predominance of non-arboviral transmission, due to contaminated aerosols (*e.g.*, from contact with livestock, in particular in case of miscarriage, manipulation of carcasses, or ritual sacrifice).

Another important observation was the significant infection rate due to sandfly-borne viruses (3.7%). These infections accounted for the second most prevalent incidences, with a magnitude equal to that of CHIKV. This result, together with the high GMT titres observed using TOSV for neutralisation tests, is highly suggestive of the circulation in Djibouti of TOSV or a closely related virus. This is in agreement with the reference 1976 sero-survey by Tesh and collaborators [13] which identified a 3.1% seroprevalence in Djibouti (“Territory of Afars and Issas”) using a PRNT technique and the prototype *Sandfly fever Naples virus* strain. Juxtaposing 1976 and 2010 serological results indicate that viruses belonging to the Naples serocomplex have been circulating for decades in Djibouti and do not represent an emerging pathogen in the region. However, because of the limitation of serological data, a complementary study on sandfly vectors, with a subsequent TOSV virus and related virus isolation and characterization would be of confirmatory importance. In a 1995 article, Fryauff and collaborators proposed an inventory of sandflies in Djibouti [8]. In the coastal plain habitat zone (in which Djibouti city is located) the predominant phlebotomine flies were *Phlebotomus alexandri* and *Phlebotomus bergeroti*. *P. alexandri* belongs to subgenus *Paraphlebotomus* and is closely related to *P. sergenti*, which has been recently associated with TOSV in Essaouira, Morocco [41]; it therefore represents a credible potential transmission vector for viruses of the Naples serogroup. In Fryauff and collaborators' study, it was found all year long, with a peak during the cool-wet season (Jan–Feb). *P. bergeroti* is related to *P. papatasi*, a vector of viruses belonging to the Naples and the Sicilian serogroup. It has never been associated with TOSV, but is the historical vector of Naples virus and Sicilian virus, that caused huge outbreak in military corps stationed in the Mediterranean, the North African and the Middle-East theatres of operations during World War II, and also proved to circulate in the 1970's in Sudan, Ethiopia and Somalia [42]. Most individuals with specific antibodies to TOSV were living in District 1. Since sandflies occupy very focal habitats, ≤1 km from their breeding sites [43], this provides robust information for future investigations aiming at formally identifying virus(es) and vector(s) implicated.

Finally, the identification of one individual with high titre VNT antibody to the Alkhumra virus extends the potential distribution area of the virus. This probable constitutes the first suspected autochthonous case in the horn of Africa. Contamination of humans may occur following the bite of ticks (*e.g.*, *Ornithodoros savignyi*, Hyalomma *dromedarii* but also as a consequence of non-arboviral transmission (*e.g.*, after manipulating carcass of infected animals or drinking contaminated raw milk [44]–[46]. A case-control study in Najran, Saudi Arabia, identified animal contact, neighbouring farms, and tick bites in the multivariate modelling whereas univariate analysis retrieved that contact with domestic animals, feeding and slaughtering animals, handling raw meat products, drinking unpasteurised milk, and being bitten by a tick were associated with Alkhumra virus infection [47]. This seropositive case deserves further investigations to clarify the epidemiological risk factors of infection in Djibouti. The lack of complementary information on the subject, such as her travel history and seropositivity profile of ambient host vectors or animals constitute obvious limitations and a follow up investigation will be necessary to complement our data findings.

In conclusion, our work strongly suggests autochthonous circulation of arboviral pathogens in Djibouti city, consistent with the past entomological and virological studies done in the same study area [5], [9], [15] in which some of the investigated pathogens, such as DENV, WNV, had been isolated and confirmed to be in circulation. The impact of *Aedes*-borne viruses (DENV and CHIKV) was found to be significant and therefore recommended a reinforcement of vector control in urban areas. We also confirmed that the exposure to *Culex*-borne viruses (WNV) was at low rate but deserves a sustained surveillance because of its epidemic potential. Though sandfly- and tick-borne viruses have never been isolated and described previously, this study provides evidence for their circulation(*i.e.* risk of exposure) in Djibouti and advocates for further investigations that would characterise and discern their vectors and their ecological cycles. Overall, the evidence adduced here are resourceful for the Djibouti Citys' Health Department in an effort to customise arbovirus prevention and control programs, that would build on the gains made by the *Roll Back Malaria Program* [48].

## Supporting Information

S1 Table Predictors of multiple seropositivity to 3 arboviruses (YF, RVF and TOSV), among Djibouti city residents in winter of 2010.
(PDF)

S2 Table Univariate analyses of subject and household factors' as predictors of arbovirus sero-positivity among Djibouti city residents in winter of 2010.
(PDF)

S1 Checklist STROBE checklist.
(DOC)
