# Supplementary material for: A Sero-epidemiological Study of Arboviral Fevers in Djibouti, Horn of Africa
Source: PLoS Negl Trop Dis. 2014 Dec 11;8(12):e3299. doi: 10.1371/journal.pntd.0003299 (PMC4263616; doi:10.1371/journal.pntd.0003299)
Supplement: S1 Table — Predictors of multiple seropositivity to 3 arboviruses (YF, RVF and TOSV), among Djibouti city residents in winter of 2010. (PDF) [file pntd.0003299.s001.pdf]

Table S1

Subject and houshold factors	Measure of association *aOR(95% CI)	pvalue
Seropositivity to YF, TOSV and RVF		
Age group: ≤19 yo	**11,9 (1,4 - 100,5)**	**0,0230**
Gender: women	6,1 (0,7 - 51,8)	0,0980
Ethnic: Afar	**6,5 (1,3 - 33,1)**	**0,0240**
Large family: 6 or more persons	5,3 (0,6 - 44,5)	0,1270
Seropositivity to TOSV and RVF		
Gender: women	3,2 (0,7 - 15,5)	0,1530
Age group: ≤19 yo	**7,7 (1,5 - 40,4)**	**0,0160**
Residential District: 2	**4,8 (1,0 - 21,7)**	**0,0430**
Large family: 6 or more persons	**6,9 (1,2 - 39,2)**	**0,0280**
Ethnic: Afar	4,7 (0,9 - 23,6)	0,0580
Seropositivity to TOSV and YF		
Gender: women	3,3 (0,7 - 16,1)	0,1470
Ethnic: Afar	4,0 (0,8 - 19,1)	0,0800
Large family: 6 or more persons	3,3 (0,7 - 16,3)	0,1410
Age group: ≤19 yo	**6,4 (1,3 - 31,9)**	**0,0240**
Seropositivity to RVF and YF		
Gender: women	2,7 (0,5 - 13,6)	0,2310
Age group: ≤19 yo	4,5 (0,9 - 23,2)	0,0710
SES level: Upper	3,5 (0,7 - 17,9)	0,1330
Large family: 6 or more persons	6,4 (0,8 - 52,7)	0,0840
Ethnic: Afar	3,9 (0,8 - 18,7)	0,0880

*adjusted odd ratio

NOTE: Factors with statistical significant association (at p<0.05) are **bolden**
