# Supplementary material for: A Sero-epidemiological Study of Arboviral Fevers in Djibouti, Horn of Africa
Source: PLoS Negl Trop Dis. 2014 Dec 11;8(12):e3299. doi: 10.1371/journal.pntd.0003299 (PMC4263616; doi:10.1371/journal.pntd.0003299)
Supplement: S2 Table — Univariate analyses of subject and household factors' as predictors of arbovirus sero-positivity among Djibouti city residents in winter of 2010. (PDF) [file pntd.0003299.s002.pdf]

Table S2:

Subject and household factors	DENV<br>OR 95% CI	TBE<br>OR 95% CI	WNV<br>OR 95% CI	YF<br>OR 95% CI	CHIK<br>OR 95% CI	TOSV<br>OR 95% CI	RFV<br>OR 95% CI
Age group: ≤19 years	0,8 (0,6 - 1,1)	1.7 (0.2 - 12.1)	0.6 (0.1 - 5.4)	2,3 (0,7 - 7,26)	0.9 (0.4 - 2.1)	1,5 (0,7 - 3,1)	**5,2 (1,5 - 18,0)***
20-39years	ref	n	1.2 (0.2 - 8.8)	0.6 (0.2 - 2.1)	1,94 (0,88 - 4,3)	ref	ref
40-59 years	0,8 (0,5 - 1,2)	5.6 (0.8 - 39.6)	1.9 (0.2 - 18.6)	0.5 (0.1 - 4.0)	n	1,0 (0,3 - 3,0)	3,0 (0,6 - 14,5)
≥60 years	1,3 (0,8 - 2,3)	n	n	n	2.3 (0.6 - 9.4)	0,9 (0,1 - 6,9)	n
Gender: women	0,8 (0,6 - 1,1)	0,8 (0,1 - 5,84)	2.4 (0.3 - 23.3)	1,5 (0,5 - 4,68)	0,6 (0,27 - 1,3)	1,4 (0,7 - 2,9)	1,0 (0,4 - 2,5)
Family: small ≤3 persons	ref	n	ref	ref	ref	ref	ref
medium (4 or 5 persons)	**1,8 (1,3 - 2,3)*****	n	4,4 (0,4 - 48)	1,64 (0,4 - 7,27)	0,7 (0,2 - 2,7)	1,2 (0,5 - 2,5)	2,6 (0,9 - 7,5)
large (≥6 persons)	**1,4 (1,0 - 2,0)***	n	3,5 (0,2 - 56)	**4,3 (1,2 - 16,0)***	**4,2 (1,8 - 10,0 ****	1,2 (0,5 - 3,1)	**3,5 (1,1 - 10,6)***
Children in household: (0)	ref	n	n	ref	ref	ref	ref
few(≤3)	**0,7 (0,5 - 0,9)****	n	n	1,13 (0,3 - 4,33)	0,6 (0,2 - 1,4)	1,6 (0,6 - 4,2)	3,4 (0,8 - 14,8)
more(4 ≥)	1,0 (0,6 - 1,5)	n	n	2,14 (0,4 - 12,6)	1,1 (0,3 - 3,8)	2,1 (0,6 - 7,4)	4,8 (0,8 - 28,2)
Ethnicity: Afar	1,3 (0,9 - 1,8)	n	n	1.9 (0.4 - 8.5)	2,0 (0,83 - 5,0)	1.0 (0.3 - 3.1)	1.8 (0.5 - 5.9)
Arab	0,8 (0,6 - 1,2)	n	1.5 (0.17 - 15.1)	1.0 (0.2 - 4.3)	1,0 (0,3 - 3,4)	1.3 (0.6 - 3.0)	2.2 (0.9 - 5.7)
Ethiopia	0,7 (0,4 - 1,5)	n	n	n	2,2 (0,5 - 9,6)	1.2 (0.3 - 4.9)	1.0 (0.1 - 7.5)
Migrants	1,0 (0,3 - 2,6)	**19.6 (2.2 - 176.9)***	n	n	3,4 (0,5 - 24,8)	n	n
Somalis	ref	1.8 (0.19 - 17.6)	1.8 (0.2 - 17.9)	0.9 (0.3 - 2.7)	ref	0.9 (0.5 - 1.8)	0.5 (0.2 - 1.1)
SES Level: low	1,0 (0,6 - 1,6)	5.4 (0.6 - 52.2)	n	0,21 (0,0 - 2,27)	0,34 (0,11 - 1,2)	1,3 (0,3 - 4,5)	0,6 (0,2 - 2,2)
middle	ref	n	n	9,53 (0,9 - 104)	ref	ref	ref
upper	1,3 (0,8 - 2,2)	0.3 (0.0 - 3.2)	n	10,1 (1,3 - 80,6)	0,36 (0,1 - 1,2)	1,3 (0,4 - 4,5)	0,5 (0,2 - 1,8)

NOTE: Bold face crude odd ratio(OR) indicates a statistical significant association; with * for pvalue of 0.05 to ≤ 0.0051, ** for pvalue of 0.005 to ≤ 0.00011, and *** for ≤ 0.0001

A letter "n" represents an association that could not be estimated due to data limitation

Table S2

……Continuation

Occupation and literacy factors	DENV<br>OR 95% CI	TBE<br>OR 95% CI	WNV<br>OR 95% CI	YF<br>OR 95% CI	CHIK<br>OR 95% CI	TOSV<br>OR 95% CI	RFV<br>OR 95% CI
Occupation: ≤13years	1,1 (0,7 - 1,7)	n	13.6 (1.9 - 94.5)*	3,44 (0,3 - 37,3)	1,0 (0,2 - 4,2)	0,9 (0,2 - 3,8)	n
Employed	0,7 (0,4 - 1,0)	1,4 (0,1 - 15,1)	1.6 (0.2 - 15.6)	5,5 (1,0 - 29,7)*	0,2 (0,0 - 1,5)	0,7 (0,2 - 2,2)	1.3 (0.4 - 3.9)
Jobless	ref	ref	0.4 (0,0 - 3.5)	ref	ref	ref	0.2 (0.1 - 0.7)*
Student	0,6 (0,4 - 0,8)*	0,8 (0,1 - 8,8)	n	3,9 (0,8 - 20,2)	0,7 (0,3 - 1,7)	1,2 (0,5 - 2,5)	4,1 (1,6 - 10,2)**
Education: ≤5years	0,8 (0,4 - 1,4)	n	6.1 (0.6 - 57.1)	2,0 (0,2 - 21,7)	n	0,7 (0,1 - 5,3)	0,0 (0,0 - 0.0)***
Illiterate	0,7 (0,5 - 0,9)*	0,5 (0,0 - 7,4)	2.8 (0.4 - 19.4)	0,4 (0,0 - 4,2)	0,4 (0,1 - 1,3)	0,8 (0,3 - 2,4)	0,4 (0,0 - 4,2)
Basic education	ref	n	1.2 (0.1 - 12.2)	ref	1.7 (0.7 - 4.0)	ref	ref
Bac+	0,8 (0,5 - 1,2)	n	n	1,7 (0,2 - 11,7)	0.7 (0.2 - 2.8)	1,1 (0,3 - 3,9)	1,7 (0,2 - 11,7)
Working from outdoors:	0,7 (0,4 - 1,4)	n	n	n	1,8 (0,3 - 9,5)	1,0 (0,2 - 4,8)	1,2 (0,1 - 11,2)
Working from indoors:	0,7 (0,4 - 1,3)	1,6 (0,1 - 17,6)	3,4 (0,2 - 53)	5,0 (0,9 - 29,5)	0,8 (0,1 - 7,4)	0,8 (0,2 - 3,9)	4,5 (1,0 - 19,5)*
**Residential environ factors**							
Residential District: 1	2,2 (1,6 - 3,0)***	n	5.3 (0.6 - 51.0)	1.8 (0.6 - 5.4)	9,6 (2,3 - 40,8)**	2,1 (1,0 - 4,6)*	1,2 (0,4 - 3,1)
2	ref	5.1 (0.5 - 48.6)	n	1.7 (0.6 - 5.3)	ref	ref	ref
3	0,9 (0,6 - 1,5)	n	1.2 (0.1 - 11.7)	n	0,9 (0,1 - 9,4)	0,4 (0,1 - 1,7)	0,7 (0,2 - 2,8)
4	1,4 (0,7 - 2,5)	n	n	n	3,2 (0,3 - 34,6)	1,4 (0,3 - 6,3)	0,9 (0,1 - 7,3)
Living nearby river bank:	1,7 (1,1 - 2,7)*	6,8 (0,7 - 64,2)	n	n	1,2 (0,2 - 9,0)	n	n
Living nearby dumpsite:	0,5 (0,3 - 0,8)*	n	n	0,5 (0,1 - 3,7)	n	2,0 (0,9 - 4,6)	2,2 (0,8 - 6,2)
Living nearby food market:	0,5 (0,3 - 0,6)***	1,3 (0,2 - 9,42)	0,7 (0,1 - 7,4)	0,5 (0,1 - 1,7)	0,1 (0,0 - 0,6)*	0,8 (0,4 - 1,6)	1,0 (0,4 - 2,5)
Living nearby Vegetable market:	0,6 (0,4 - 0,8)**	2,4 (0,3 - 17,1)	0,8 (0,1 - 7,8)	0,2 (0,0 - 1,7)	n	0,8 (0,4 - 1,8)	0,9 (0,3 - 2,4)
Living nearby abbattoir:	0,4 (0,3 - 0,6)***	2,8 (0,4 - 19,7)	n	0,3 (0,0 - 2,0)	0,1 (0,0 - 1,0)*	1,0 (0,4 - 2,1)	1,0 (0,4 - 2,8)
Living nearby open sewage:	0,2 (0,0 - 1,5)	n	n	n	n	1,3 (0,2 - 9,0)	4,7 (1,2 - 18,9)*
Sleeping out in open at night:	1,7 (1,2 - 2,4)**	n	4,2 (0,4 - 40)	n	3,6 (1,4 - 9,2)*	0,9 (0,2 - 3,5)	1,5 (0,4 - 6,4)
Keeping animal(s):	1,6 (1,2 - 2,2)**	1.7 (0.2 - 2.0)	2,6 (0,2 - 29)	n	0,3 (0,0 - 2,3)	0,6 (0,2 - 1,9)	0,7 (0,2 - 3,0)
Exposure to birds:	n	n	n	n	n	n	n

NOTE: Bold face crude odd ratio(OR) indicates a statistical significant association; with * for pvalue of 0.05 to ≤ 0.0051, ** for pvalue of 0.005 to ≤ 0.00011, and *** for ≤ 0.0001

A letter "n" represents an association that could not be estimated due to data limitation
